# MIRA Rehab Exergames for Older Male Residents in a Care Home Center in Saudi Arabia: Protocol for a Feasibility Randomized Controlled Trial

**DOI:** 10.2196/39148

**Published:** 2022-12-20

**Authors:** Mohammad Zougar, Chris Todd, Lisa McGarrigle, Emma Stanmore

**Affiliations:** 1 Department of Physical Therapy Faculty of Applied Medical Sciences Taif University Taif city Saudi Arabia; 2 School of Health Sciences Faculty of Biology, Medicine & Health The University of Manchester Manchester United Kingdom

**Keywords:** exergame, balance, older adults, telerehabilitation, feasibility, elderly care, aging, elderly population, rehabilitation, virtual therapy, digital rehabilitation, physical activity

## Abstract

**Background:**

Physical activity leads to improvements in morbidity, mortality, and quality of life, especially when it is progressive, challenging, and regular. There is strong evidence that strength and balance exercises decrease the risk of falling. However, traditional exercises may be tedious and not very motivating for participants. Exergames have been found to increase engagement and enjoyment for older users.

**Objective:**

This study will conduct a feasibility randomized controlled trial (RCT) on the use of MIRA Rehab Exergames among older male residents in a care home setting in Saudi Arabia. A sample of 30 eligible participants will be recruited to meet feasibility study requirements.

**Methods:**

We will recruit 38 residents in the care home who will be randomly allocated to either an intervention or a control group. The intervention participants will perform gamified exercises using the MIRA telerehabilitation platform (30 minutes 3 times per week for 6 weeks). The control group will receive educational advice based on booklets of the Otago exercise program and be encouraged to exercise (30 minutes 3 times per week for 6 weeks). Participants will be assessed at weeks 0, 6, and 12. Assessments will include feasibility measures (eligibility, recruitment and attrition rates, and practicalities of data collection methods) and participant outcome measures (balance, strength, mobility, adherence, quality of life, fear of falling, depression, acceptability, and usability).

**Results:**

Data collection started in November 2021 and ended in March 2022. The study is currently in the data analysis stage, which commenced in May 2022. The findings from this feasibility RCT will be used to design a definitive RCT to test whether the MIRA Rehab Exergame program benefits older people in Saudi Arabia who may not like participating in traditional exercise programs and may be unwilling or unable to leave their homes.

**Conclusions:**

This study will contribute to our understanding of how to recruit in this specific population and provide information to inform the design of a future RCT.

**International Registered Report Identifier (IRRID):**

DERR1-10.2196/39148

## Introduction

### Background

A fall is defined by the Prevention of Falls Network Europe group as “an unexpected event in which the participants come to rest on the ground, floor, or lower-level” [1]. Approximately 27% of older people who fall 3 or more times per year are transferred to emergency departments, while 20%-30% of older fallers sustain injuries that cause movement difficulties [2]. In addition to negative consequences such as higher morbidity levels, decreased activity, poor quality of life, and early nursing home admission, falls have been associated with increased death rates [3]. Many older adults who fall also experience notable fear of falling, and their activity levels can be reduced by up to 40% [4]. Almegbel et al [5] reported in a cross-sectional study (with a sample size of 1182 individuals) that 49.9% of the older population (aged 65 years and older) of Saudi Arabia experiences a fall every year. Moreover, the United Nations has reported that nearly 5.5% of the population of Saudi Arabia were aged 60 years or older by 2017, and that by 2050, the proportion of people aged over 65 years in the population will rise to 23% [6]. The average life expectancy in Saudi Arabia is 73.5 years for males and 76.5 years for females, with an overall life expectancy of 74 years, and increasing healthy longevity is of importance [7]. To prevent falls and their negative consequences, evidence-based physical activity such as strength and balance training needs to be implemented and adhered to [8,9]. However, physical activity engagement among older people remains low, particularly among those living in relatively lower-income neighborhoods. Older adults may be encouraged to expand their activities if others influence them, expenses are kept low, and enjoyment is high, which can increase self-efficacy for exercise [10].

### Physical Activities

Previous studies have found that physical inactivity leads to higher morbidity and mortality rates, while physical activity improves quality of life [11]. Furthermore, systematic reviews have concluded that exercise is the best single intervention for fall prevention, especially when it is progressive, challenging, and regular [8,12]. Balance training requires both strength and balance components, which increase stability [13]. Successful exercise plans are differentiated by various features, including whether multiple static and dynamic stability tasks are adapted to the risk level of older adults, whether balance training increases in challenge over time, and whether it is carried out with minimal assistance. In addition, resistance exercise training that progresses over time needs to be included. Appropriate programs should include strategies to encourage long-term behavior change in older adults as well as fall prevention components [14].

According to Al-Hazzaa and Al-Marzooqi [15], while the benefits of exercise are well recognized, it remains a great challenge to encourage inactive people to start participating in exercise programs. Brumels et al [16] suggested that traditional exercises may be tedious and not very motivating for the participants. A lack of interest in workouts may contribute to lower-than-expected adherence [16]. Furthermore, rapid urbanization, extreme weather, cultural hurdles, a lack of social support, the absence of an efficient physical activity program, and a lack of time and resources can all make physical activities a difficult decision for Saudis [17]. Therefore, Saudis need to develop ways to make exercise practice more engaging, available, and accessible in educational institutions, clinics, workplaces, and communities.

### Novel Technology

Exergames are video games that blend gaming and physical activity with animation. When Exergames include therapy-based exercises, they can be a form of telerehabilitation [18]. They can also include virtual reality simulations [19]. Exergames are considered to be a feasible way of enhancing engagement and removing barriers to training, leading to improvements for older people [19]. Exergames can be designed to cover a wide variety of conditions. Furthermore, in the case of some systems, the types of exercises and the difficulty level of the program can be prescribed by qualified physiotherapy professionals. The MIRA Rehab Exergames system has been codeveloped with older adults. The gamified exercises are based on the Falls Management Exercise (FaME) and Otago exercise programs [20,21], which have been shown to reduce falls in older people [18]. MIRA is an exergaming software product designed for medical use, and it has been Conformité Européenne–certified as a class I medical device [22]. It complies with UK National Health Service safety standards for patient data protection and privacy [22]. To date, this technology has not been used in Saudi Arabia.

### Aims and Objectives

The main aim of this study was to conduct a feasibility randomized controlled trial (RCT) using MIRA Rehab Exergames among older adults in Saudi Arabia. Feasibility was assessed in terms of participant recruitment rates, intervention delivery, intervention acceptability (barriers and facilitators), 6-week follow-up attrition rates, and data collection and evaluation processes. Feasibility was also assessed in terms of the suitability of the outcome measures (balance, physical function, lower limb strength, depression, fear of falling, quality of life, and adherence among older adults). Intervention acceptability included factors relating to the acceptability, viability, and usability of MIRA Rehab among older adults, either positively or negatively (ie, barriers and facilitators to use). This study did not assess the effectiveness of the intervention. However, such information is required before conducting a definitive future RCT. As this is a feasibility study, it was underpowered to assess the effectiveness of the intervention. However, preliminary results in relation to outcomes are presented.

## Methods

### Study Design

The study was conducted in Saudi Arabia and was a single-center, 2-arm, single-blind, parallel-group feasibility RCT. The reporting of this feasibility RCT followed the Consolidated Standards of Reporting Trials extension for feasibility trials in order to offer a clear report and appraisal [23] (see Multimedia Appendix 1).

### The Study Setting: A Care Home Center

The trial was undertaken at the Social Care Home for the Elderly (the Dar Al-rieayat Al-aijtimaeia social care home) in Makkah, Saudi Arabia, which is a care home center for older male adults. This is the only center in Makkah providing specialist services for older males, and it is funded by the government. The residential home center provides care for any male citizen who is aged 60 years or older and is unable to conduct his own affairs. It offers care for patients referred from hospital and for those who have no families. The aim of this center is to provide care for citizens who are aged 60 years and older and are unable to live independently. Residents are required to be registered as free from infectious and mental health conditions [24]. Such governmental homes and residential care facilities for older people provide social, medical, and psychological care. Their services vary, but they generally facilitate cultural, professional, recreational, and sports activities [24]. For cultural reasons, as the researcher is male, we were unable to recruit females. Women receive residential care in separate facilities away from the main center (the Dar Al-rieayat Al-aijtimaeia center).

### Participant Recruitment

To enable participant identification and recruitment in this residential care setting, the involvement of professional staff was required in the care setting.

### Meeting With Professionals

The aim of the meeting with professionals was to confirm the arrangements for identifying potential participants (older adults) on the basis of the professionals’ knowledge, expertise, and records. The researcher needed to interact with health care providers, including physiotherapists, rehabilitation specialists, nurses, social workers, physicians, and other health workers responsible for caring for older people at the center. These professionals provide basic medical services and ensure the physical and psychological well-being of the residents. Seeking assistance from the staff was crucial for the feasibility of the study. During the meeting, the researcher described the study (but not the specific hypotheses), focusing on the study’s eligibility criteria (see below). The researcher then asked the professionals to use the eligibility criteria to screen for eligible participants.

### Participants

Potential participants (male residents at the care home center who meet the eligibility requirements) were contacted by the health care professionals to assess their ability and willingness to take part in the study. The health care professionals then provided each potential participant an invitation letter, a participant information sheet, and a consent form. As some residents may be illiterate or have difficulty reading, verbal descriptions were provided as necessary. After a period of 2-3 days, potential participants were contacted in person by the health care professionals to confirm that they are willing to take part in the study and to obtain their consent. Only after obtaining consent, they were approached by the researcher.

### Inclusion and Exclusion Criteria

The inclusion and exclusion criteria for this feasibility study are outlined in Textbox 1.

**Inclusion and exclusion criteria.**

**Inclusion criteria**

- Older adults aged 60 years and older (60 years is the retirement age in Saudi Arabia)
- Resident at the participating care home center for older adults
- Ability to communicate in Arabic
- Ability to provide informed consent
- Ability to walk 9.1 m (30 ft) with or without a supportive aid
- Ability to participate physically
- Medically stable (based on patient medical notes)

**Exclusion criteria**

- Deaf, registered blind, or severe visual or auditory problems identified from medical records
- Use of medications that induce sleep, fatigue, dizziness, or drowsiness (eg, antihistamines, antidepressants, anxiety medications, cancer treatments, and some antihypertensive medications)
- History of severe mental health problems
- Uncontrolled movements owing to neurological disorders (eg, Parkinson disease, ataxia, and cerebral palsy)
- Severe middle ear problems, vestibular problems, or severe vertigo or dizziness
- Fracture within 6 months
- Severe cardiovascular problems (eg, deep vein thrombosis) or unstable diastolic blood pressure
- Recent surgery within 6 months
- Unwilling to participate

### Sample Size

As this is a feasibility study that does not intend to assess effectiveness, demonstration of differences between groups is not a key objective. We are interested in the parameters of the outcome measurements in order to assess the utility of each outcome measure and enable sample size estimation for a future definitive trial. Thus, the focus is on the acceptability and usability of the MIRA Rehab program, and we shall use the results to guide the design of a potential definitive RCT [25]. As recommended by Hooper [26], the total number of participants was approximately 30. Anticipating an attrition rate of 20% for feasibility study, we recruited 19 persons in each arm to recruit approximately 15 older participants in each group [26].

### Randomization

Randomization to the intervention or control group was undertaken by a separate member of the research team after baseline measurements were taken. We used an equal allocation ratio of 1:1. Taking into account the allocation ratio for 2 groups, the block size can be 2, 4, or 6. Randomization was undertaken by permuted blocks of size 2-6, using the Sealed Envelope randomization service [27].

### Intervention

**Figure 1:**
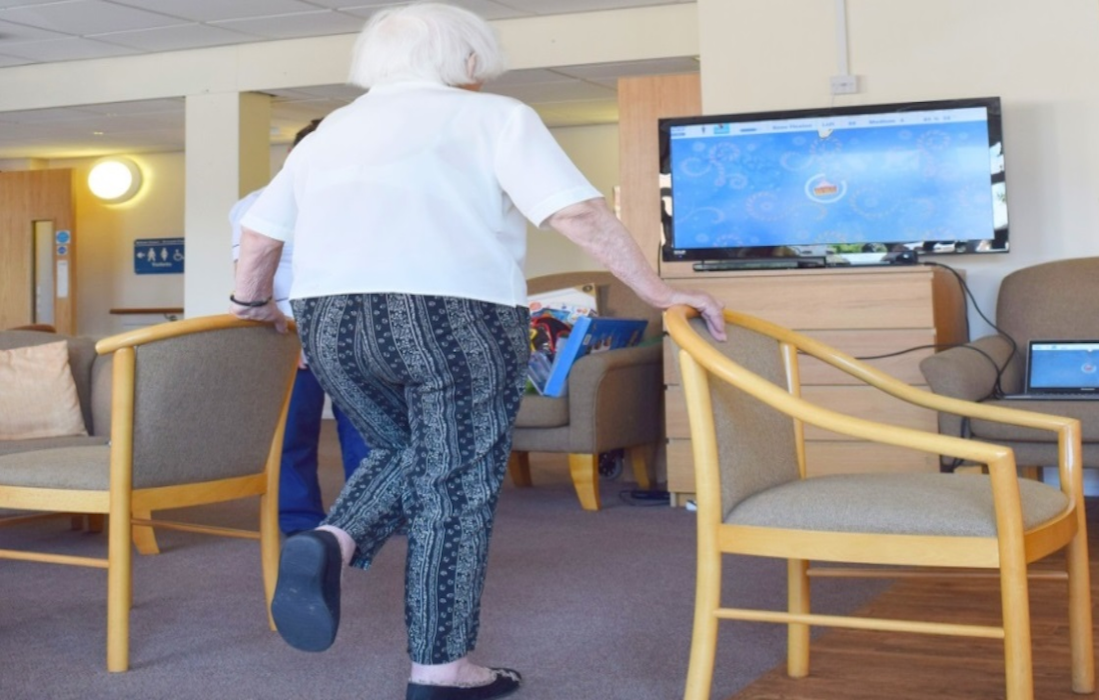
A participant in the United Kingdom performing single-leg support using MIRA Rehab Exergame (reproduced with permission of author ES).

Consistency of measurement for the MIRA Rehab intervention is based on the trajectories of human skeletal joints [28] (Figure 1). This system has been developed to provide a way of performing physiotherapy activities using avatars, live motion tracking, direct feedback, and an intelligent environment. The MIRA Rehab Exergames keep track of the clients’ achievements (eg, speed, score, and number of games played). They have been developed with and for older people with support from the University of Manchester and fall prevention therapists [22].

**Figure 2:**
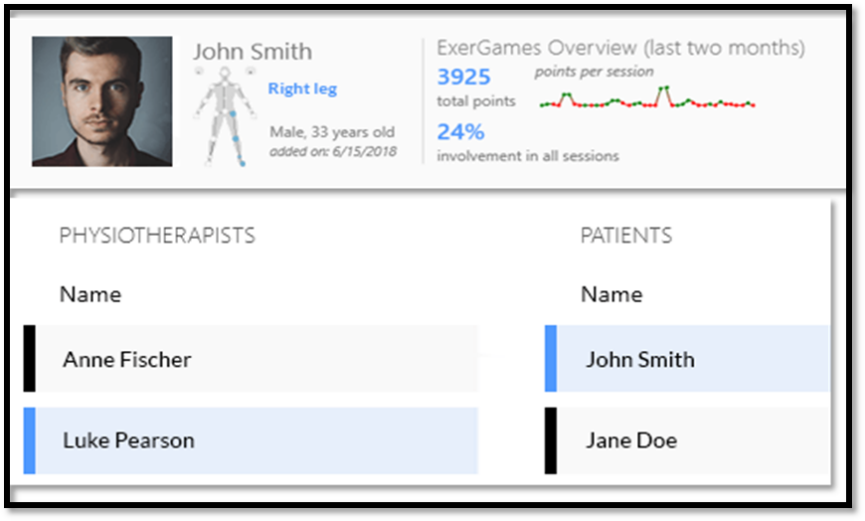
Example of an individual account and patient assignment screen view (MIRA Rehab website).

**Figure 3:**
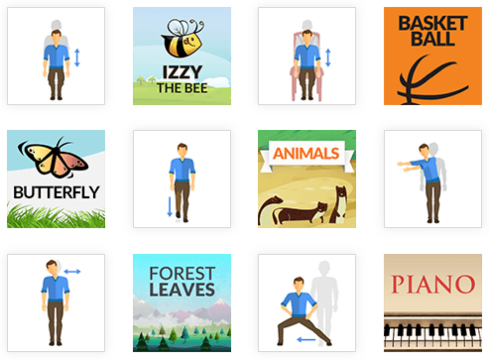
Example of a variety of games and exercises, which can be personalized on the basis of patients' needs and game preferences (MIRA Rehab Exergame).

MIRA Rehab Exergames provides feedback on correct movements and tracking features (eg, range of movement) to a wide number of patients and health professionals who interact with them. They can create patient files and assign them to clinicians who can remotely monitor progress if required (Figure 2). The choice of Exergame can be made and adapted on the basis of clinical recommendations and patient preferences (Figure 3). However, certain exercises can be adapted to improve balance, strength, and mobility, such as sitting to standing, squatting, forward leaning from the standing position, leg extension from the seating position, and reaching the upper extremities in multiple directions. The choice of Exergames in the study was based on the participants’ preference for colors, game play, and music. For patients who are not familiar with MIRA Rehab Exergames, video tutorials are provided. Furthermore, it is possible to personalize the Exergame software and set practical personal objectives to facilitate improvement [29].

### Preparation and Setup

For preparing the MIRA Rehab Exergame, the room must be at least 2×2 m to accommodate the equipment and space needed to exercise. The training room that was used is a part of the indoor physiotherapy clinic located at the care home center that is only available during daytime. The MIRA Rehab software (installed on a laptop computer) and a transportable Microsoft Kinect sensor with a 3D motion-sensor camera (version 2; Microsoft Corp) were connected to a TV screen. Each session started with calibration so that the users could correctly position themselves for optimal performance and efficient motion tracking. The lead investigator (MZ) and assistants explained to the participants how to perform the exercises [18]. 

### Participants’ Files

A participant file could be created for each individual using the MIRA Rehab software. Participant files record all the data from MIRA Rehab regarding each participant’s activities and status (ie, selected Exergames, completed games, uncompleted games, game modifications, and game scores). Each file stores this information together for each individual for ease of reference during the treatment plan. To create individual accounts and establish files for the participants, certain information is needed (eg, name or identifier, age, date of birth, weight, and height). All the physical activities conducted by participants were saved on the MIRA Rehab platform drive, which is protected by a username and password and accessed only by the main researcher. The participants’ involvement in the sessions was represented as a percentage indicating adherence to the exercises. Adherence was presented as the number of attended classes or sessions.

### Duration

The participants participated in 30-minute sessions that were repeated 3 times a week for 6 weeks. Participants did not perform the required physical activities for the study during the next 6 weeks’ follow-up. However, they continued their regular physical therapy sessions.

### Safety and Support

The Exergame program was conducted and directed under the supervision of a physiotherapist to ensure participants’ safety. For additional participant support and safety reasons, a shock absorber mat, 2 supportive chairs, and a parallel bar facing the TV screen at a distance of 2 m was required in the Exergame room (training room). Additionally, the physiotherapists observed the participants during the exercise session. The nurses also continuously checked the participants for any changes in health during the exercise sessions. Warming up exercises were provided to avoid possible muscle soreness and pain. It was explained to the participants that muscle soreness, pain, and fatigue are a natural physiological responses to exercise.

### Tailoring the Fall Prevention Strategy of MIRA Rehab Exergames

The participants were initially assessed by the physiotherapist, who then adapted the program in accordance with individual participants’ needs and abilities. Next, the physiotherapists selected the most suitable Exergames from a list based on Otago/FaME traditional exercises. The duration of each Exergame, the duration of the rest periods, and the number of exercises were tailored to the individual’s level and rehabilitation needs. Since the aim of the study is to assess the feasibility of the intervention for improving physical abilities, selected games were used on the basis of specific movements.

### Control Group

In this study, all the participants received an educational package provided by physiotherapists (30 minutes 3 times per week for 6 weeks) to the control group in addition to their usual care. They continued their usual activities. These included their normal exercise, recreation, and television watching as well as other aspects of their everyday routine at the care home center. The researcher explained and demonstrated the educational materials to staff in one of the meeting rooms at the center, making sure that the staff agreed that the educational materials were suitable for the older adults at the center. The materials are available in English and Arabic, and the physiotherapists provided these materials to the participants as verbal instructions based on the FaME and Otago exercise programs [20,21]. The participants performed these exercises at the physiotherapy department or at their rooms if they could do it independently. The educational material involved chair-based exercises, and it was available at the Later Life Training website [30] (see Multimedia Appendices 2 and 3).

### Outcome Measures

This feasibility study included all the quantitative and qualitative outcome measures we anticipate, including in the future RCT, to inform the design of the main study. The outcome measures were assessed for their suitability for the main trial. These were assessed in terms of practicality (ie, recruitment rates, attrition rates, and eligibility criteria), data collection methods, required resources, and adherence (see Textbox 2).

Other variables of interest for this trial were as follows: (1) balance (measured by the Berg Balance Scale) [31]; (2) functional ability (balance, functional ability, and mobility measured by the Timed Up and Go test) [32]; (3) outcomes on the Functional Reach Test [33,34]; (4) the Short Physical Performance Battery [35]; (5) fear of falling (measured with the Fall Efficacy Scale–International) [36]; (6) depression (measured using the Geriatric Depression Scale) [37]; (7) quality of life (measured using the EQ-5D-5L) [38]; (8) exercise adherence (measured through the MIRA Rehab platform); and (9) usability and acceptability (measured using the System Usability Scale and the Technology Assessment Model) [39]. We also collected qualitative structured interview data from the participants who were asked to describe their experiences, including what they liked (facilitators) and disliked (barriers), to help us understand the success (or otherwise) of the intervention (Multimedia Appendix 4 and Table 1).

**Feasibility outcomes.**

**Recruitment and eligibility (no criteria set):**

- Participants were identified through medical records. The participants were screened on the basis of the eligibility criteria.
- Participants who do not meet the eligibility criteria.
- Participants who do not participate and their reasons for not participating.

**Data collection (no criteria set):**

- Participants who completed the 6-week sessions (intervention group).
- Participants who completed the assessments.

**Attrition (no criteria set):**

- The number of participants who did not complete the 6-week sessions (intervention group).
- Participants who did not complete the assessments.

**Resources (no criteria set):**

- The time required to complete the questionnaires.
- The time required to perform the physical assessments.
- The time required to complete the intervention.

**Adherence (no criteria set):**

- The participants who attended each Exergame session in accordance with the MIRA Rehab system (based on the participants’ logging in or logging off).

**Table 1:** Schedule of outcome measurements.

Outcome measure	Week 0 (baseline)	Week 6 (follow-up 1)	Week 12 (follow-up 2)
Balance (Berg Balance Scale)^a^	✓	✓	✓
Functional ability (Timed Up and Go)^a^	✓	✓	✓
Functional Reach Test^a^	✓	✓	✓
Short Physical Performance Battery^a^	✓	✓	✓
Fear of falling (Fall Efficacy Scale–International)^a^	✓	✓	✓
Depression (Geriatric Depression Scale)^a^	✓	✓	✓
Quality of life (EQ-5D-5L)^a^	✓	✓	✓
Exercise adherence (through the MIRA Rehab platform)^a^	Daily	Daily	Daily
Usability (intervention group)^a^			✓
Acceptability (intervention group)^a^			✓
Structured interview^b^	✓	✓	

^a^ Quantitative outcome measures.

^b^Qualitative outcome measures.

### Blinding

A physiotherapist (independent from the investigator) performed the baseline assessments for all the older participants (before allocation) during the first week. Then, an independent member of the team allocated the participants to different groups using a random number generator and informed the main investigator. The second assessment in week 6 was performed by another physiotherapist or physician (different from the initial one who performed the baseline measurements). The third and final assessment at week 12 was performed by another physiotherapist or physician who was not familiar with the previous assessments. While we attempted to maintain blinding at follow-up, this was not possible. Double blinding was not possible, as the participants knew whether they were exercising using the MIRA Rehab system. It was not possible to blind the medical team and the participants because the participants received rehabilitation sessions as part of their daily routines.

### Statistical Analysis

Statistical analysis is currently underway. The baseline characteristics of the participants will be reported using mean and SD values for normally distributed variables and median and IQR values ranges for nonnormally distributed variables. Analysis is being conducted using SPSS (version 27.0; IBM Corp) [40]. Continuous data, such as retention and recruitment rates, will be identified as percentages. Both groups will be compared at baseline with respect to their continuous and categorical variables using independent samples *t* tests and the Fisher exact test, respectively. Changes in balance and function will be compared between the intervention group and the control group from baseline (week 0) to follow-up (weeks 6 and 12). Inferential findings will be interpreted cautiously, as the feasibility analysis is underpowered for detecting significant effects. Distribution of data will be explored as normal or skewed. For normal distributions, mean and SDs will be used. For skewed distributions, minima, maxima, medians, and quartiles will also be used. We will report 95% CIs for unadjusted differences in mean outcome scores between the groups. Changes in mean scores over 3 time points (weeks 0, 6, and 12) as well as changes in average scores for each result will be recorded using repeated measures ANOVA. The effect size for the differences between the intervention and control groups and the variations within the intervention group will be estimated. Other relevant models will be used in the case of nonnormally distributed data. The findings will be used to calculate the optimum sample size (number of participants) for determining a statistically significant true effect in preparation of a future definitive RCT. Both intention-to-treat and per-protocol analyses will be undertaken. This feasibility RCT is a hypothesis-generating study for preparing and conducting additional analyses not identified in this protocol paper.

### Ethics Approval and Research Governance

This study has been approved by the University of Manchester Research Ethics Committee (reference number 2021-11191-20154). To enable access to participants at the social care home for older individuals, permission has also been obtained from the local institution in Saudi Arabia that is responsible for the investigator’s research and training. This study adheres to the tenets of the Helsinki Declaration. Prior to participation in the study, all participants were given a written informed consent form.

## Results

This study is funded by Saudi Arabian cultural mission as part of a PhD project. Data collection started in November 2021 and ended in March 2022. The study is currently in the feasibility data collection stage, which commenced in May 2022. Additional qualitative data collection and analysis are ongoing and will be completed in January 2023.

## Discussion

### Expected Findings

This is the first feasibility RCT of MIRA Rehab Exergames in a residential care center for older adults (aged 60 years and older) in Saudi Arabia. While other researchers have studied various Exergame technologies, they have not examined purposely designed Exergame technology for older adults. Exergames for fall prevention were administered for over 6 weeks with a 6-week follow-up and compared to a control group receiving an exercise educational package. The study tested the feasibility of conducting an RCT of this novel technology in a new cultural context. This feasibility study protocol is intended to provide as much information as possible for a future randomized controlled trial. The study investigates the delivery of MIRA Rehab Exergames in the context of Saudi Arabia and included a small sample, with one site researcher. The findings may also inform whether Arabic and Saudi adaptations should be added to provide an adapted Exergame system that reflects cultural considerations. It will be critical for future research to examine how Exergames affect larger populations. Researchers should also carry out longitudinal investigations to assess changes in fall rates. Additionally, researchers should evaluate optimal exercise doses to inform potential future implementation.

### Conclusions

This study will contribute to our understanding of how to recruit in this specific population and provide information to inform the design of a future RCT. MIRA Rehab Exergames may be a feasible and practical exercise intervention for enhancing the functional performance of older adults and to improve physical activity levels. Health care providers, such as physiotherapists and rehabilitation teams, may include Exergames such as the MIRA Rehab programs as part of a comprehensive physical therapy intervention for older adults living in care homes or similar settings.

## Appendix

Multimedia Appendix 1 CONSORT 2010 checklist for reporting pilot or feasibility trials.

Multimedia Appendix 2 The Arabic version of chair based training (CBT) provided by Later Life Training.

Multimedia Appendix 3 The English version of chair based training (CBT) provided by Later Life Training.

Multimedia Appendix 4 Participants’ observations during data collection.

## Abbreviations

FaME: Falls Management Exercise
RCT: randomized controlled trial

## Data Availability

All data generated during this study and included in this published article and its supplementary information files are available upon request. For further details, pleases contact MZ (mohammad.zougar@postgrad.manchester.ac.uk).
