# Supplementary material for: MIRA Rehab Exergames for Older Male Residents in a Care Home Center in Saudi Arabia: Protocol for a Feasibility Randomized Controlled Trial
Source: JMIR Res Protoc. 2022 Dec 20;11(12):e39148. doi: 10.2196/39148 (PMC9812269; doi:10.2196/39148)
Supplement: Multimedia Appendix 4 [file resprot_v11i12e39148_app4.docx]

**Participants’ observation during data collection**

**Multimedia appendix (4): The topic Guide**

**The use of MIRA Rehab Exergames to improve physical abilities for older adults at a care home centre in Makkah city. A feasibility randomised controlled study (structured interview for observational purposes)**

1. **Interview preparation**

- The interview will be done at the physiotherapy department at the care home centre. The place is usually quiet and empty during the lunch time. The place is naturally lightened during this time of the day. Each participant will be informed in advance that he will be asked a serious of questions about the study at the beginning of the first and final weeks).
- The researcher will prepare a comfortable chair for the participant and ask if he needs a soft drink or water.
- Each participant will be asked structured questions and their responses written down directly
- The researcher will undertake a structured interview with participants in Arabic (below reproduced in Arabic language and English)
- The researcher will remind the participants that they have consented to participate in the data collection and complete scales and assessments with records kept on paper.

1. **Structured interview**

| **English Version** | **Yes/نعم** | **No/لا** | **Comments/تعليقات** | **النسخة العربية** |
| --- | --- | --- | --- | --- |
| Do you think the use of exercises based on electronic games is useful? |  |  |  | هل تعتقد استخدام التمارين المبنية على الالعاب الالكترونية كانت مفيدة؟ |
| Have you been told about the effect of exercise by physiotherapists? |  |  |  | هل تم اخبارك عن تأثير التمارين الرياضية من قبل اخصائيين العلاج الطبيعي؟ |
| Do you suggest adding modifications to the program? Number if yes?  (Final interview only) |  |  |  | هل تقترح اضافة تعديلات للبرنامج؟ عدد اذا الاجابة نعم؟ |
| Do you prefer to exercise traditionally or through exercises based on electronic games?  (Final interview only) |  |  |  | هل تفضل ممارسة التمارين بشكل تقليدي او عن طريق التمارين المبنية على الالعاب الالكترونية؟ |
| Were the instructions clear when using the program?  (Final interview only) |  |  |  | هل كانت التعليمات واضحه عند استخدام البرنامج؟ |
| Did you encounter any problems when using the device?  (Final interview only) |  |  |  | هل واجهتك أي مشاكل لدى استخدامك للجهاز؟ |
| Is there a variety of exercises offered?  (Final interview only) |  |  |  | هل يوجد تنوع في التمارين المقدمة؟ |
| Has the intensity of the exercises been adjusted for you?  (Final interview only) |  |  |  | هل تم تعديل شدة التمارين من أجلك؟ |
| Did you face any difficulties during the exercise?  (Final interview only) |  |  |  | هل واجهت أي صعوبات اثناء التمرين؟ |
| Do you wish to use the device in the future?  (Final interview only) |  |  |  | هل تتمنى استخدام الجهاز في المستقبل؟ |
